# Ethnozoological study of traditional medicinal appreciation of animals and their products among the indigenous people of Metema Woreda, North-Western Ethiopia

**DOI:** 10.1186/s13002-018-0234-7

**Published:** 2018-05-23

**Authors:** Fasil Adugna Kendie, Sileshi Andualem Mekuriaw, Melkamu Andargie Dagnew

**Affiliations:** Department of Biology, College of Science, Bahir Dar University, P.O. Box 79, Bahir Dar, Ethiopia

**Keywords:** Traditional medicine, Indigenous knowledge, Ethnozoology, Zootherapy

## Abstract

**Background:**

Using animals for different purposes goes back to the dawn of mankind. Animals served as a source of food, medicine, and clothing for humans and provided other services. This study was designed to undertake a cross-sectional ethnozoological field survey among the residents of Metema Woreda from November 2015 to May 2016.

**Methods:**

Data were collected through studied questionnaires, interviews, and focus group discussions with 36 purposively selected respondents.

**Results:**

Ethnozoological data were collected of the local name of the animals, part of the animal used, mode of preparation and administration, and of additional information deemed useful. A total of 51 animal species were identified to treat around 36 different ailments. Of the animals used therapeutically, 27 species were mammals, 9 were birds, 7 arthropods, 6 reptiles, and 1 species each represented fish and annelids. Furthermore, the honey of the bee *Apis mellifera* was used to relieve many ailments and scored the highest fidelity value (*n* = 35.97%). The snake (*Naja naja)* and the teeth of crocodiles (*Crocodylus* spp.) had the lowest fidelity value (*n* = 2.56%).

**Conclusion:**

The results show that there is a wealth of ethnozoological knowledge to be documented which could be of use in developing new drugs. Hence, it is hoped that the information contained in this paper will be useful in future ethnozoological, ethnopharmacological, and conservation-related research of the region.

## Background

Using animals for different purposes goes back to the dawn of mankind. Animals served as a source of food, medicine, and clothing for humans and provided other services [1]. The traditional medicinal knowledge of indigenous people across the globe has played an important role in identifying living organisms which are endowed with medicinal values important for treating human and livestock health problems. Since ancient times, animals and their products have been used in the preparation of traditional remedies in various cultures [2]. Human societies have accumulated a vast store of knowledge about animals through the centuries, which is closely integrated with many other cultural aspects, and this zoological knowledge is an important part of our human cultural heritage [3].

The cure for human ailments using therapeutics from animals is known as zootherapy [4]. It plays a significant role in the healing practices, magic rituals, and religious societies all over the world [5, 6]. In the modern era, zootherapy constitutes a major alternative among many other known therapeutic practices in the world. Wild as well as domestic animals and their by-products such as hooves, skins, bones, feathers, and tusks serve as important ingredients in the preparation of curative, protective, and preventive medicines [5, 7, 8].

Traditional medicines have been important in connection with drugs like digitoxin, reserpine, tubocurarine, and ephedrine [9]. Of the 252 essential chemicals that have been selected by the World Health Organization, 8.7% come from animals [10].

Loss of traditional knowledge of indigenous communities had impact the development of modern medicine. It is important to document the traditional knowledge of human communities, since the majority of such communities are losing their socioeconomic and cultural characteristics [10]. Animals and the products derived from their body organs constitute part of the inventory of medicinal substances which are used widely by the people since time immemorial, and such practices still exist in traditional medicines [10]. Traditional healing methods involving hundreds of insect and other invertebrate species are reviewed by Meyer-Rochow [11]. In South Africa, animals and plants are commonly used as traditional medicines for both the healing of ailments and for symbolic purposes such as improving relationships and attaining good fortune [12].

In Traditional Chinese Medicine, more than 1500 animal species had been recorded to be some medicinal use [13]. In Brazil, Alves and Rosa reported the medicinal use of 283 animal species for the treatment of various ailments [14–17].

In Ethiopia, 70% of human and 90% of livestock population depend on traditional medicine. Although Ethiopians are known for their widespread use of traditional medicines with various levels of sophistication within the indigenous medical lore, the vast knowledge of the traditional uses of animal species of therapeutic value is not well documented for the various regions of the country. Moreover, since most of the knowledge is conveyed along generations through word of mouth, the traditional knowledge as well as the products used by these people is under threat [18].

In Metema Woreda, there were a number of studies about ethnobotany and traditional medicine, diversity, and floristic compositions of plants. However, despite the great diversity of ethnic groups and cultures in this area, ethnozoological studies of traditional medicinal animals have not yet been sufficiently addressed. Metema Woreda is characterized by the presence of a mosaic of ethnic groups with deep rooted culture of using traditional medicinal plants and animals. Hence, this study is aimed to explore ethnozoology and preparations of animals and its products as traditional medicine used to cure different human and animal ailments.

## Methods

### Study area description

The study was conducted in Metema Woreda in the Amhara National Regional State. The Woreda is about 333 km to the North West of Bahir Dar, the Capital City of Amhara Regional State. Metema is one of the Woredas in the Semien Gondar Zone, bordered by Qwara in the south, Sudan in the west, Mirab Armachiho in the north, Tach Armachiho in the northeast, Chilga in the east, and Takusa in the southeast. The Woreda constitutes a total of 20 Peasant Kebele administrations, of which 18 are rural-based peasant administration areas [19, 20]. The Woreda is the home of many ethnic groups including Agaw, Tigrie, Oromo, Gumuz, and Amhara migrated from the different angles of the country for different reasons displaying a diversity of cultures and indigenous belief.

### Selection of study sites

A preliminary study was conducted in November 2015 to select specific study sites in the Woreda and test data collection tools. The study was conducted in six kebeles of Metema Woreda (Birshign; Kokit; Mender 6, 7, and 8; Metema Yohannis; Aftit; and Meka) from November 2015 to May 2016. These kebeles were purposively selected based on the availability of many traditional healers, presence of different ethnic groups, and accessibility of the area.

### Sampling and data collection

The ethnozoological data (local name of animals, mode of preparation and administration, and part of the animal used) were collected through questionnaires, interviews, and focus group discussion with selected residents of Metema Woreda. Purposively, 36 key informants were selected, and questionnaires, interviews, and focus group discussion were made within these informants [21]. These informants were local herbalists, traditional healers, farming experts, midwives, and spiritual intellectuals. The selections of key informants were based on their experience and recognition as knowledgeable members concerning traditional zootherapeutics (the so called expert by the local people) [22]. Different types of ethnozoological data were collected from each type of key informants.

### Group discussion

Brief group discussions were made at each site prior to the distribution of detailed questionnaires on the importance of animals in traditional medicine and related issues with the selected informants of the study site. During the discussions, an attempt was made to encourage the healers in such a way that their cooperation would be of benefit to the country and at same time an informed consent was obtained before data collection.

### Semi-structured interviews

A semi-structured checklist and interview questions were prepared in advance. The interviews were based on this checklist, and some issues were raised promptly depending on the responses of an informant. The interview was held in Amharic, the language of the people by the researchers. The place and time for the discussion was set based on the interest of the informants.

### Informant consensus

During the course of the study, each informant was visited three times in order to confirm the reliability of the ethnozoological information. Consequently, the responses of an informant that were not in harmony with each other were rejected since they were considered as unreliable information.

### Animal specimen collections and identifications

The local names and associated attributes of medicinal animals were recorded for each of the species. The specimens with its common name, photograph, dead skin, hair, fur, and some products were collected and taken to Bahir Dar University (BDU) for species identification. Identification of the medicinal animals was done in BDU, using Internet and animal key by comparison with collected plates and illustrations.

### Data analysis

The data obtained were summarized and analyzed using descriptive statistical methods. In the ethnozoological data that were obtained from the interviews on reported medicinal animals and associated knowledge, fidelity level (FL) was calculated as the percentage of respondents claiming the use of a certain animal species for the same ailments, for the most frequently reported diseases or ailments as

$$ \mathrm{FL}\ \left(\%\right)={\mathrm{Np}}^{\ast }\ 100/N $$

where *Np* is the number of respondents that claim a use of a species to treat a particular disease and *N* is the number of respondents that use the animals as a medicine to treat any given disease [23]. The range of fidelity level (FL) is from 1 to 100%; high values indicate that this particular animal species is used by large number of people, while a low value shows that respondents disagree on the usefulness of a species in treating ailments.

## Results

This study revealed the traditional medicinal knowledge of treating various kinds of ailments using different animals and their parts/products by local inhabitants of different kebeles of Metema Woreda (North-Western Ethiopia). Many people were found to lack formal schooling education, but they have knowledge about the use of local animal resources for traditional medicines.

Socio-demographic characteristics of the respondents such as sex, age, educational level, and marital status were collected and presented (Table 1).

**Table 1:** Socio-demographic characteristics of the respondents

Basic information	Number of respondents	Percentage (%)
Sex		
Male	34	94.4
Female	2	5.6
Age		
35–44 years	6	16.7
45–60 years	20	55.5
> 60 years	10	27.8
Educational level		
Illiterate	15	41.7
Literate	21	58.3
Marital status		
Married	34	94.4
Single	1	2.8
Divorced	1	2.8

Information regarding the way to acquire traditional medicinal knowledge, duration of time to use traditional medicine, the reason that forces the people to use traditional medicines, categories of people that use traditional medicine, the outlooks of people about the use of traditional medicine, conservation, and documentation mechanisms of traditional medicinal animals were gathered from all respondents (Table 2).

**Table 2:** Information that was acquired by close-ended questionnaire

No.	Questions	Choices	No. of respondents	Percentage (%)
1	Where did you learn traditional medicinal knowledge?	A) Family	16	44.4
		B) Books	4	11.1
		C) Surrounding society	12	33.3
		D)Experience	4	11.1
		Total	36	
2	How many times people use traditional medicines?	A) Sometimes	15	41.7
		B) Always	13	36.1
		C) Situational	8	22.2
		Total	36	
3	What was the reason that forces the people to use traditional medicines?	A) Economy	7	19.4
		B) Lack of modern medicine	10	27.8
		C) Effectiveness	19	52.8
		Total	36	
4	Which categories of people use traditional medicines in large quantity?	A) Ethnic group	5	13.9
		B) Nations	1	2.8
		C) Religion	5	13.9
		D) All	25	69.4
		Total	36	
5	What looks like the outlooks of people about use of traditional medicines?	A) Good	15	41.7
		B) Bad	1	2.8
		C) Intermediate	20	55.5
		Total	36	
6	Are there any conservation and documentation mechanisms of traditional medicinal animals?	A) Yes	3	8.3
		B) No	31	86.1
		C) Some	2	5.5
		Total	36	

Fifty-one animal species (Table 5) were found to be used for the treatment of over 36 kinds of ailments. There were 27 species belonging to mammals, 9 to birds, 7 arthropods, 6 reptiles, and 1 each among the fish and annelid (Table 3).

**Table 3:** Animal groups and number of species used for traditional medicine in the study area

No.	Animal groups	Number of species	Percentage (%)
1	Mammals	27	52.9
2	Birds	9	17.6
3	Reptiles	6	11.8
4	Fish	1	2
5	Arthropods	7	13.7
6	Annelid	1	2

The animals and their parts/products were found to be used for the treatment of around 36 different kinds of ailments including rheumatism, malaria, wart, stomachache, toothache, herpes, headache, rabies, tuberculosis, anemia, trachoma, gastritis, asthma, paralysis, and cough. The animals were used as whole or their products like milk, blood, organ, meat, teeth, and honey for the treatment of various ailments (Table 8).

According to the data (Table 4), meat/fat was the most widely used medicinal parts/products of animals in traditional medicine, followed by visceral organs, products and bone/teeth, and external body parts with similar percentages. On the other hand, an animal’s whole body and excreta, and blood were found to be the least used medicinal parts/products of animals.

**Table 4:** Animal parts or products used to traditional medicine in the study area

No.	Medicinal parts/products of animals	No. of parts/products used	Percentage (%)
1	Meat/fat	23	23.5
2	Visceral organ (liver, spleen, Bile, stomach/intestine)	21	21.4
3	Products (honey, venom, milk, butter)	13	13.3
4	Bone/teeth	12	12.2
5	External Body part (head, tail, leg, skin, horn, spine/thorn)	12	12.2
6	Excreta (stool and urine)	6	6.1
7	Whole body	6	6.1
8	Blood	5	5.1

In the study area, different parts or products of animals were used to treat different types of ailments. The highest number of cow parts or products 8 (3.8%) used to treat 8 (4.5%) ailments. The second rank was occupied by common warthog (*Phacochoerus africanus*), porcupine (*Hystrix* spp.), spotted hyena (*Crocuta crocuta*), and elephant (*Elephas maximus*) with similar number of parts/products 5 (2.5%) and used to treat 8 (4.5%), 13 (7.5%), 11 (6.2%), and 7 (3.9%) ailments, respectively (Table 5).

**Table 5:** Mode of application/administrations of traditional medicines

No.	Mode of application	No. of application	Percentage (%)	Mode of entry
1	Eating	30	28.0	Oral
2	Drinking	27	25.2	Oral
3	Tying	18	16.8	Not enter
4	Anointing	14	13.1	Dermal
5	Banding	6	5.6	Dermal
6	Massaging	6	5.6	Dermal
7	Fumigation	3	2.8	Nasal
8	Heating	3	2.8	Dermal

Preparations varied according to ailment and involved cooking, burning, crushing/grinding, wrapping, powdering, and drying or the use of fresh animal parts/products (Table 6).

**Table 6:** Medicinal animals and their parts/products used and number of ailments treated

Animal group	Common name	Local name	Scientific name	No. of parts/products used	No. of ailments treated
				*N* (%)	*N* (%)
Mammals	Wild boar	Ria	*Sus scrofa*	1 (0.5)	4 (2.2)
	Common warthog	Kerkero	*Phacochoerus africanus*	5 (2.5)	8 (4.5)
	Cow	Lam	*Bos taurus*	8 (3.9)	8 (4.5)
	Cheetah	Aboshemane	*Acinonyx jubatus*	1 (0.5)	1 (0.6)
	Camel	Gimel	*Camelus dromedaries*	1 (0.50	4 (2.2)
	Porcupine	Jart	*Hystrix* spp.	5 (2.5)	13 (7.3)
	Human	Sew	*Homo sapiens*	1 (0.5)	1 (0.6)
	Donkey	Ahiya	*Equus africanus asinus L.*	1 (0.5)	5 (2.8)
	Rat	Ayti	*Rattus* spp.	3 (1.5)	3 (1.7)
	Spotted hyna	Gib	*Crocuta crocuta*	5 (2.5)	11 (6.2)
	Gazelle	Agazen	*Gazella* spp.	2 (1.0)	2 (1.1)
	Goat	Fiyel	*Capra aegagrus hircus* L.	4 (2.0)	12 (6.7)
	Hippopotamus	Gumare	*Hippopotamus amphibius*	1 (0.5)	3 (1.7)
	Pigs	Asama	*Sus scrofa domesticus*	2 (1.0)	3 (1.7)
	Monitor lizard	Arjano	*Varanus* spp.	1 (0.5)	1 (0.6)
	Sheep	Beg	*Ovis aries*	1 (0.5)	1 (0.6)
	Olive baboon	Zingero	*Papio anubis*	3 (1.5)	4 (2.2)
	Cat	Dimet	*Felis domesticus*	1 (0.5)	1 (0.6)
	Elephant	Zihon	*Elephas maximus*	5 (2.5)	7 (3.9)
	Bear	Dib	*Melursus ursinus*	1 (0.5)	1 (0.6)
	Vervet monkey	Tota	*Chlorocebus pygerythrus*	1 (0.5)	2 (1.1)
	Common fox	Kebero	*Canis* spp.	2 (1.0)	5 (2.8)
	Giraffe	Kechinie	*Giraffa camelopardalis*	2 (1.0)	1 (0.6)
	Dog	Wusha	*Canis familaries*	1 (0.5)	1 (0.6)
	Ethiopian hare	Tinchel	*Lepus fagani*	3 (1.5)	4 (2.2)
	Groundhog	Shikoko	*Marmota monax*	1 (0.5)	1 (0.6)
	Bat	Yelelit wof	*Cynopterus sphinx*	1 (0.5)	2 (1.1)
Birds	Vulture	Timb ansa	*Gyps* spp.	2 (1.0)	2 (1.1)
	Pigeon	Ergib	*Columba livia*	1 (0.5)	3 (1.7)
	Duck	Dackye	*Duck* spp.	1 (0.5)	1 (0.6)
	Ostrich	Segon	*Struthio camelus*	3 (1.5)	3 (1.7)
	Hen	Dero	*Gallus gallus domesticus*	3 (1.5)	4 (2.2)
	Osprey	Gedie	*Pandion haliaetus*	1 (0.5)	2 (1.1)
	Erckel’s francolin	Koki	*Pternistis erckelii*	2 (1.0)	2 (1.1)
	Red billed oxpecker	Arechi	*Buphagus erythrorhynchus*	1 (0.5)	1 (0.6)
	Bald eagle	Chilat	*Haliaeetus leucocephalus*	1 (0.5)	1 (0.6)
Reptiles	Snake	Ebab	*Naja naja*	3 (1.5)	6 (3.4)
	Crocodile	Azo	*Crocodylus* spp.	3 (1.5)	5 (2.8)
	Python	Zendo	*Python* spp.	4 (2.0)	7 (3.9)
	Tortoise	Ali	*Testudo graeca*	1 (0.5)	2 (1.1)
	Chamaleon	Esist	*Chamaeleo chamaeleon*	1 (0.5)	1 (0.6)
	Lizard	Enshilalit	*Lacertilia* spp.	1 (0.5)	2 (1.1)
Fish	Fish	Assa	Any fish spp.	2 (1.0)	2 (1.1)
Arthropods	Scorpion	Ginti	*Palamnaeus swammerdami*	1 (0.5)	1 (0.6)
	Bees	Nib	*Apis mellifera*	2 (1.0)	13 (7.3)
	Termite (Queen)	Mist	All spp.	1 (0.5)	1 (0.6)
	Field cricket	Fenta	*Gryllus campestris*	1 (0.5)	1 (0.6)
	Gnat (small insect)	Tinign	All spp.	1 (0.5)	3 (1.7)
	Bomble bee	Tinziza	*Bombus* spp.	1 (0.5)	3 (1.7)
	Ticks	Meziger	All tick spp.	1 (0.5)	1 (0.6)
Annelid	Leeches	Alekit	All spp.	1 (0.5)	1 (0.6)

The traditional medicines were administrated via different modes. Eating, followed by drinking, tying, anointing, banding and massaging and, fumigation and heating were the major modes of application (Table 7). Solids and liquids were administered orally, whereas banding, heating, anointing, and massaging materials were applied to the skin. Medicinal fumes were allowed to enter the body via the nose, while some parts of animals like bones, skin, and teeth were believed to serve a healing purpose by tying them on the neck or other parts of the body. Most of the remedies did not involve the addition of substances like sugar, water, butter, honey, teff and millet flour, salt, spice, milk, egg, and coffee, but there were cases in which such additives were used.

**Table 7:** Methods of preparation of traditional medicinal in the study area

No.	Types of preparation	No. of preparation	Percentage (%)
1	Fresh	40	36.4
2	Cooking	26	23.6
3	Burning	15	13.6
4	Crushing/grinding	8	7.3
5	Wrapping	8	7.3
6	Powdering	7	6.4
7	Drying	6	5.4

Fidelity levels (FL) demonstrate the percentage of respondents claiming the use of a certain animal or its product for the same ailments. The honey of bee species (*Apis mellifera*) used to relieve wart, asthma, diarrhea, throat pain, stomachache, cough, and tuberculosis had the highest FL (*n* = 35, 97%) followed by meat of wild boar (*Sus scrofa*) to treat rheumatism, syphilis, stomachache, and malaria (*n* = 32, 89%), milk of goat (*Capra aegagrus hircus*) to treat eye disease, gastritis, headache, measles, tuberculosis, vomiting, and rheumatism (*n* = 27, 75%), teeth of the common warthog (*Phacochoerus africanus*) to treat toothache, wart, and rheumatism (*n* = 26, 72%), meat of the porcupine (*Hystrix* spp.) to treat swelling, tuberculosis, headache, AIDS, asthma, rheumatism, and gastritis (*n* = 24, 67%), and urine of Gazelle (*Gazella* spp.) to treat urination problems (*n* = 23, 64%). On the other hand, biles of common fox (*Canis* spp.) to cure eye problem and toothache (*n* = 2, 5.6%), the upper skin of the snake (*Naja naja)* to cure headache (*n* = 2, 5.6%), and the teeth of crocodile (*Crocodylus* spp.) to cure epilepsy (*n* = 2, 5.6%) have the lowest fidelity level value (Table 8).

**Table 8:** Medicinal animals, parts/products used, and their fidelity level

Animal group	Common name	Scientific name	Parts/product used	Ailments treated	No. of respondents claimed (*n*)	Fidelity level (FL)	Mode of applications
Mammals	Wild boar	*Sus scrofa*	Meat	Rheumatism, syphilis, stomachache, and malaria	32	89	Eating
	Common warthog	*Phacochoerus africanus*	Teeth	Swelling, toothache, wart and rheumatism	26	72	Heating
			Blood	Malaria, asthma, and rheumatism	12	33	Drinking
			Skin	Herpes	4	11	Anointing
			Bile	AIDS	8	22	Drinking
			Horn	Swelling	4	11	Heating
	Cow	*Bos taurus*	Butter	Malaria and paralysis	8	22	Eating
			Milk	Rabies and TB	18	50	Drinking
			Urine	Malaria	4	11	Drinking
			Spleen	Anemia, malaria and trachoma	13	36	Eating
			Omasum	Gastritis	4	11	Eating
			Liver	Anemia	9	25	Eating
			Blood	Wart	10	28	Drinking
	Cheetah	*Acinonyx jubatus*	Skin	Hemorrhage	3	8	Tying
	Camel	*Camelus dromedarius*	Milk	Headache, rheumatism, malaria and diarrhea	20	56	Drinking
	Porcupine	*Hystrix* spp.	Meat	Swelling, TV, headache, AIDS, asthma, rheumatism, gastritis, and hypertension	24	67	Eating
			Bile	Asthma/diabetes, stomach scramble	11	31	Drinking
			Stomach/intestine	Diarrhea and diabetes	7	19	Eating
			Thorn/spine	Wound and broken leg	14	39	Tying
			Liver	Diabetes disease	3	8	Eating
	Human	*Homo sapiens*	Stool	Wart	3	8	Anointing
	Donkey	*Equus africanus asinus L.*	Milk	Measles, cough, trachoma/rabies, and internal problem	22	61	Drinking
	Rat	*Rattus* spp.	Meat	Intestinal disease	5	14	Eating
			Foot	Nightmare	4	11	Tying
			Blood	Wart	6	17	Anointing
	Spotted hyna	*Crocuta crocuta*	Bone	Epilepsy and bad spirit	12	33	Tying
			Skin	Protection from evil eye and during labor	9	25	Tying
			Bile	Erythroblastosis and nightmare	8	22	Tying
			Liver	Infection of skin	5	14	Banding
			Skin	For communicable diseases and bad spirit	11	31	Tying
			Meat	For swollen sex organ, epilepsy and anemia	5	14	Eating
	Gazelle	*Gazella* spp.	Urine	For urination problem	23	64	Drinking
			Bile	Syphilis		0	Drinking
	Goat	*Capra aegagrus hircus* L.	Milk	Eye disease, gastritis, wound, headache, measles, TB, eye disorder, vomiting, snake poison, and rheumatism	27	75	Drinking
			Fat	Wound and Toothache	16	44	Banding
			Liver	Trachoma	7	19	Massaging
			Butter	Headache and ear infection	8	22	Massaging
	Hippopotamus	*Hippopotamus amphibius*	Bone	Breast swelling, sunburn, and body fracture	6	17	Banding, drinking
	Pig	*Sus scrofa*	Meat	Rheumatism and headache	4	11	Eating
			Blood	Skin infection	4	11	Anointing
	Monitor lizard	*Varanus* spp.	Skin	Infant communicable disease	6	17	Tying
	Sheep	*Ovis aries*	Milk	Malaria	4	11	Drinking
	Olive baboon	*Papio anubis*	Hind skin/skin	Broken/misplaced bone and wound/burning	9	25	Tying
			Meat	Rabies prevention for dogs and HIV/AIDS	13	36	Eating
			Bile, meat	AIDS	6	17	Eating, drinking
	Cat	*Felis domesticus*	Skin	Spiritual problem	3	8	Tying
	Elephant	*Elephas maximus*	Bile	Kidney failure	3	8	Drinking
			Bone	Herpes and diarrhea	6	17	Massaging
			Ivory	Herpes	3	8	Anointing
			Urine	Herpes, urination disorder	3	8	Drinking
			Skin	Herpes, back pain, skin wound, and trachoma	6	17	Anointing
	Bear	*Melursus ursinus*	Bile	Epilepsy	4	11	Drinking
	Vervet monkey	*Chlorocebus pygerythrus*	Meat	For STDs, anemia for children	3	8	Eating
	Common fox	*Canis* spp.	Brain tissue and meat	Epilepsy, mental disorder	4	11	Eating/drinking
			Bile	Toothache, eye problem, and internal problem	2	5.6	Drinking
	Giraffe	*Giraffa camelopardalis*	Urine and milk	TB	3	8	Drinking
	Dog	*Canis familaries*	Bone	Epilepsy	3	8	Tying
	Ethiopian hare	*Lepus fagani*	Excreta	Soars/wound	4	11	Anointing
			Meat	Cattle disorder, epilepsy	8	22	Fumigation, drinking
			Fat	Wart	5	14	Anointing
	Groundhog	*Marmota monax*	Meat	For coughing and fattening baby	7	19	Eating
	Bat	*Cynopterus sphinx*	Meat	Hepatitis, mental disorder	21	58	Eating
Birds	Vulture	*Gyps* spp.	Leg	Epilepsy	3	8	Fumigation
			Meat	Mental disorder	4	11	Eating
	Pigeon	*Columba livia*	Meat	Mental disorder, body fracture, and heart failure	12	33	Eating
	Duck	*Duck* spp.	Meat	TB	4	11	Eating
	Ostrich	*Struthio camelus*	Meat and egg	Muscle strain and broken bone and paralysis	4	11	Massaging, anointing
	Hen	*Gallus gallus domesticus*	Whole body	For physical injury and wound	9	25	Drinking
			Liver and fat	Swelling wound, pneumonia	16	44	Eating
	Osprey	*Pandion haliaetus*	Bone	Epilepsy, body fracture	5	14	Tying
	Erckel’s francolin	*Pternistis erckelii*	Meat	Internal problem	3	8	Eating
			Bile	STDS	3	8	Drinking
	Red billed Oxpecker	*Buphagus erythrorhynchus*	Blood	Skin fungus	4	11	Anointing
	Bald eagle	*Haliaeetus leucocephalus*	Blood	Skin fungus	4	11	Anointing
Reptiles	Snake	*Naja naja*	Coat	Headache	2	5.6	Tying
			Venom	Malaria and snake bite	4	11	Anointing
			Head	Diarrhea, evil eye, and headache	6	17	Tying
	Crocodile	*Crocodylus* spp.	Bile	Coughing, TB, teeth rheumatism	4	11	Drinking Anointing
			Bone	Communicable disease	3	8	Tying
			Teeth	Epilepsy	2	5.6	Tying
	Python	*Python* spp.	Bone	Rabies and swelling	3	8	Tying and Banding
			Tail and bone	Cancer and swelling	3	8	Banding
			Fat	Wound and ear disease	7	19	Banding,
			Meat	Rabies, foot crack, and ear disorder	13	36	Eating, anointing
	Tortoise	*Testudo graeca*	Teeth	Swelling	3	8	Heating
			Shell	Trypanosomiasis, nose bleeding	6	17	Fumigation
	Chameleon	*Chamaeleo chamaeleon*	Whole body	Cancer, body fattening	6	17	Tying
	Lizard	*Lacertilia* spp.	Whole body	Dry cough and anemia	3	8	Drinking
Fish	Fish	Any fish spp.	Meat	Rheumatism	4	11	Eating
			Bile	Eye disorder	3	8	Eating
Arthropods	Scorpion	*Palamnaeus swammerdami*	Meat	Scorpion bite	6	17	Massaging
	Bee	*Apis mellifera*	Honey	Wart, asthma, diarrhea, throat pain, stomachache, cough, TB, mumps, heart failure	35	97	Eating, drinking
			Larvae	Stomach disorder	3	8	Drinking
	Termite (Queen)	All spp.	Whole body	Fattening of livestock	3	8	Eating
	Field cricket	*Gryllus campestris*	Whole body	Eye disease	3	8	Eating
	Gnat (small insect)	All spp.	Honey	Stomachache, eye disorder, and coughing	13	36	Eating
	Bumble bee	*Bombus* spp.	Honey	Coughing, malaria, and stomachache	3	8	Eating
	Ticks	All tick spp.	Blood	Fungal disease on the skin	3	8	Anointing
Annelid	Leeches	All spp.	Head	Rheumatism	3	8	Massaging

## Discussion

In Ethiopia, 70% of human and 90% of livestock population depend on traditional medicine [18]. In this study, 51 animal species and their products were collected and identified that were believed to be a cure/prevention of over 36 kinds of ailments. Other studies reported in Ethiopia showed that approximately 23 animals and/or their parts were identified to be used in traditional medicines in Degu tribes in Tigray region [22]. Sixteen species of medicinal animals were collected and identified for treating 18 different human ailments in the Kafta-Humera District, Northern Ethiopia [24]. The study conducted by Borah and Prasad recorded a total of 44 different species of animals which are used for the treatments of 40 different ailments [21]. In South Africa, Whiting et al. identified 147 medicinal vertebrate species representing 60 mammal species, 33 reptile species, 53 bird species and 1 amphibian species [12]. Oliveira et al. also described 23 animal species that used as traditional medicines [25]. Of a total 36 vertebrate species used in the treatment of ailments and disease, mammals comprised 50%; they were birds, fishes, reptile, and amphibians [26].

The inhabitants of the study area were found to use different parts/products of animals for the treatment of different kinds of ailments. Animals and the products derived from their body organs constitute part of the inventory of medicinal substances [10]. Meyer-Rochow also reported different organs of invertebrate animals used as traditional medicines [11].

In this study, parts/products of medicinal animals were grouped under meat/fat, blood, visceral organ, whole body, excreta, bone/teeth, and product categories and these categories were similar to ones reported by Haileselasie [22]. Other researches also stated that wild and domestic animals and their by-products such as hooves, skins, bones, feathers, and tusks are important ingredients in the preparation of curative, protective, and preventive medicine [7–9].

Preparations varied according to ailment and involved cooking, burning, crushing/grinding, wrapping, powdering, and drying [11]. In this study, egg is considered as one of the products of animals. The egg of ostrich (*Struthio camelu*) was mentioned as a traditional medicine in Table 8. It is used to treat muscle strain, broken bone, and paralysis. Gidey Yirga et al. showed medicinal animals have various methods of preparation for different types of ailments like crushing, powdering, squeezing, direct use, and cooking [27]. Haileselasie reported that animals are used as whole or body parts or by-products like milk, blood, organ, flesh, antler, and feathers for the treatments of different kinds of human ailments including cough, asthma, tuberculosis, paralysis, earache, herpes, weakness, and muscular pain [22].

This study showed that traditional medicines were administrated by drinking, eating, anointing, tying, branding, fumigation, and massaging. The study conducted by Gidey Yirga et al. showed most of traditional medicines were administrated orally and through dermal. Fumigating materials such as smokes were also entering into the body using nasal opening to treat different ailments. Some parts of animals such as bones, skin, and teeth were believed to be medicine by tying on the neck or other parts of the body [27].

The majority of the remedy preparations did not have additive substance while the remaining had different additive substances like sugar, water, butter, honey, teff and millet flour salt, spice, milk, egg, and coffee. The result of this study is similar to research conducted by Gidey Yirga et al. [27]. Haileselasie stated that many animals were used for the treatment of multiple ailments singly or in combinations with other animal products or/and plants like seeds, flowers, latex (resins in some cases), and roots [22].

The honey of bee species (*Apis mellifera*) is known to relieve wart, asthma, diarrhea, throat pain, stomachache, cough, and tuberculosis and achieves the highest fidelity level, whereas biles of common fox (*Canis* spp.) to cure eye problem and toothache, upper coats of snake (*Naja naja*) to cure headache, and teeth of crocodile (*Crocodylus* spp.) to cure epilepsy have the lowest fidelity level. On the other hand, Jaroli et al. stated that the uses of animals that are commonly known by the Garasiya informants have higher fidelity levels than less common known species [27]. He reported the cooked flesh of bat (*Cynopterus sphinx*) used to relieved cough and fever has the highest FL followed by blood of pigeon (*Columba livia*) to treat paralysis and urine of cow (*Bos taurus*) for wound healing, while the flesh of the pig (*Sus scrofa*) to relieve muscular pain and elephant (*Elephas maximus*) for pimples have the lowest fidelity level.

The finding of this study suggested that the traditional zootherapeutic remedial measures followed by the native people of Metema Woreda plays an important role in their primary healthcare. The documentation of this indigenous knowledge on animal-based medicines should be very helpful in the formulations of strategies for sustainable management and conservation of bio-resource as well as providing potential for novel drug discoveries [21].

## Conclusions

The result shows that animals and their parts/products occupy key positions in the traditional medicine and medical practices to treat different ailments. Whole bodies or parts/products of traditional medicinal animals were used as a medicine. It was obvious that the members of the local communities studied possessed considerable knowledge related to preparation, administration, parts/products used, ingredients added, and other issues of traditional remedies. However, efforts to document, conserve, and manage the indigenous knowledge and skill were very scarce, and important indigenous knowledge is getting lost together with the elders and experts. Hence, it is important to document, conserve, and manage the indigenous knowledge, and further research should be done to test the products scientifically for product development.
